# Acknowledgment to Reviewers of *JPM* in 2020

**DOI:** 10.3390/jpm11020063

**Published:** 2021-01-21

**Authors:** JPM Editorial Office

**Affiliations:** MDPI AG, St. Alban-Anlage 66, 4052 Basel, Switzerland

Peer review is the driving force of journal development, and reviewers are gatekeepers who ensure that *JPM* maintains its standards for the high quality of its published papers. Thanks to the cooperation of our reviewers, in 2020, the median time to first decision was 19 days and the median time to publication was 37 days. The editors would like to express their sincere gratitude to the following reviewers for their precious time and dedication, regardless of whether the papers were finally published:
Abasse, SoumethLin, QishanAbbas-Aghababazadeh, FarnooshLin, Shu-ChunAbreu, CarlosLin, WeifengAcedo Nunez, Maria Del PilarLin, Yen-KoAdams, SolomonLindemann, StephanAdenzato, MauroLio, DoAdonakis, GeorgeLitak, JakubAdunyah, Samuel E.Liu, HengruiAguglia, EugenioLiu, MinghuaAhmad, FahimLo Giudice, RobertoAiello, MarcoLobo, JoaoAlaimo, AlessandroLopez, Jose IgnacioAlbright, DanaLópez, Lucía MéndezAlconchel, FelipeLópez-Borrull, AlexandreAleksandrovych, VeronikaLu, QuanlongAlexander, MathewLuzzago, StefanoAliaño González, María JoséMa, EnboAl-Imam, AhmedMachicado, Jorge D.Al-Jawaldeh, AyoubMaddipati, VeerannaAlkilany, ReemMadonov, PavelAlmgren, KhaledMai, Hang NgaAl-Mubarak, Bashayer R.Maksimov, SergeyAl-Nuaimi, Ali H. HusseenMallis, PanagiotisAlsaab, HashemMammadova-Bach, ElminaAlsharhan, HindMancuso, FrancescaAlves, MarcoMandal, ChanchalAmbros, PeterManenti, AntonioAmbuj, KumarManley, Paul N.Anagnostis, AthanasiosManne, SharonAnberitha Matthews, AnberithaMannelli, LorenzoAncel, JulienManolopoulos, Vangelis G.Andereggen, LukasManukjan, GeorgiAnderson, Michael BrunManzo, CiroAndolina, Jeffrey R.Mao, XumingAndreou, EleniMarczyk, MichalAndrews, Shea J.Mariotti, AlessandroAngelova, Plamena R.Markel, ArcadyAnifandis, GeorgeMarkopoulos, Georgios SAntognelli, CinziaMarkovska, RumyanaAntony, ReubenMarrodán Serrano, María DoloresArasanz Esteban, HugoMartinelli, ChiaraArbitrio, MariamenaMartino, Tartaglia GianlucaAriani, AlaricoMary, DidierArnold, Amy C.Masarone, DanieleAsensi, VictorMaspero, CinziaAssari, ShervinMatacchione, GiuliaAtzori, LauraMatschke, JohannAudrius, DulskasMatsuda, NaoyukiAuten, Richard L.Matsumoto, NaokiAvino, AdelaidaMaturana, Evangelina López DeAvtanski, DimiterMaugeri, AndreaAwate, SanketMaurya, ShailendraAziz, AtharMayer, PhilippBadiali, GiovanniMayr, WinfriedBagiu, Iulia-CristinaMcArthur, GrantBajaj, RuchikaMcCuaig, Jeanna M.Bajek, AnnaMcKelvie, Penny A.Bakillah, AhmedMcPeck, MichaelBalogh, IstvánMeade, MelissaBandapalli, Obul ReddyMedeiros-Domingo, ArgeliaBanks, LoriMedema, Jan PaulBaran, AnnaMedina-Rodríguez, Eva M.Bargiel-Matusiewicz, KamillaMędrzycka, WiolettaBarkanyi, ZsuzsannaMehariya, SanjeetBarlow-Stewart, KristineMehta, SunaliBarosi, GiovanniMeola, GiovanniBarragán, RocioMercer, ClaireBartsch, Jörg W.Messersmith, AmyBasak, IndranilMetzinger, LaurentBeaumont, MarcMeyer, Till JasperBecciolini, AndreaMichalopoulos, Nikolaos V.Bel’skaya, LyudmilaMichikawa, MakotoBelayew, AlexandraMijailovich, SrbaBelcher, John R.Milanovic, DesankaBellia, ChiaraMilavetz, BarryBelyaev, IgorMilczarek, MałgorzataBenfante, RobertaMilenkovic, Vladimir M.Benfield, David A.Mimidis, KonstantinosBennardo, FrancescoMini, EnricoBenson, MikaelMinic, ZoranBentel, Jacqueline M.Mitra, JoyBerardelli, IsabellaModerau, NinaBerardi, Anna ConcettaMohammed Geba, KhaledBerger, SlavaMohr, ThomasBernardo, AntoniettaMolin, FredrikBernaudin, Jean-FrançoisMonastero, RobertoBernhard, WolfgangMondal, Utpal KumarBertsias, GeorgeMoniuszko, HannaBeversdorf, DavidMonji, AkiraBevilacqua, RobertaMorak-Młodawska, BeataBevilacqua, VitoantonioMoreira, PaulaBhardwaj, RajeshMorgenstern, ChristianBilski, JanMorris, KeithBittel, Adam J.Moscalu, MihaelaBocchieri, SalvatoreMu, PingBoerma, MarjanMühlhaus, TimoBoggero, IanMulherkar, ShalakaBohlmann, MichaelMüller, PatrickBonato, MatteoMullin, StephenBoonen, KurtMuniz, JuanBorawski, WojciechMunshi, SoumyabrataBourgoin, SylvainMuskin, Philip R.Boussios, StergiosMusso, NataleBowden, GregoryMusteata, MihaiBowerman, MelissaMutoh, TatsushiBradshaw, NicholasMytych, JenniferBratt, AlisonNagaraj, ViniBreitenfeld Granadeiro, LuizaNagwekar, JanhaviBrennenstuhl, HeikoNakano, MasahitoBrogna, ClaudiaNakhjavani, MaryamBrooke, JoanneNam, Ki ChangBrunetti, OronzoNaranjo-Hernández, DavidBruzzese, FrancescaNardone, ValerioBugiotti, FrancescaNarra, Hema PrasadBukhari, Marwan A.S.Naruishi, KojiBuková, AlenaNatarajan, SivaramanBurckhardt, ChristophNaud, PatriceBurdukiewicz, MichałNavarro, Jose FranciscoBussalleu, EvaNelson, Sarah M.Cacialli, PietroNevšímalová, SoňaCai, YingyunNielsen, Vance G.Caluori, GuidoNieminen, PenttiCampbell, ColleenNiemira, MagdalenaCampus, GuglielmoNikolaos, MachairasCañadas-De La Fuente, Guillermo A.Nishida, HarutoCapoccia, MassimoNishio, MizuhoCapozzi, Vito AndreaNobili, StefaniaCardinali, LucillaNowak, Paweł FryderykCarraro, UgoNowicka, GrażynaCarrasquilla, GermanNowicki, RomanCarrier, PaulNuvoli, SusannaCarrington, Jane MOancea, RoxanaCaruso, SalvatoreOchi, ShinichiroCastelo-Branco, MiguelOdland, JonCengiz, Ibrahim FatihOkuma, YusukeCerwenka, HerwigOosterwijk, EgbertCesaro, ArturoOrlacchio, ArturoChaber, RadosławOrso, EvelynChaddad, AhmadOsuna, EduardoChang, MinsunOvsenik, MajaChatterjee, SampurnaPackiam, Vignesh T.Chaudhari, BimalPadilla-Eguiluz, Norma G.Chen, Chien-ChinPaepegaey, Anne-CécileChen, Sean Chun ChangPaganelli, RobertoChen, XingchengPaglia, DavidChen, Yih-SharngPalermo, SaraCheng, LinPan, Hung-ChuanCheng, XinlaiPaoletti, GiovanniChiamulera, ChristianPapachristodoulou, AlexandrosChitale, ShalakaPapi, PieroChiu, Hua-ShengPapież, MonikaCho, HanjinPaquette, BenoitChrysanthakopoulos, Nikolaos A.Paradiso, AngeloChu, Pei-YiParalič, JánChuntao, ZhaoParnetti, LucillaCiavarella, CarmenParrish, RichardCiszewski, WojciechPastor Vicedo, Juan CarlosCiszewski, Wojciech MichałPastor-Villaescusa, BelénCiudin, AndreaPatel, Jai N.Colgan, NiallPatel, NibeditaColla, EmanuelaPatil, VeerupaxagoudaColuccia, MauroPaudel, Keshav RajContreras, Joey APaudel, PradeepCornu, CatherinePawlik, AndrzejCorris, Paul A.Pecho-Vrieseling, ElineCortini, FrancescaPedemonte-Sarrias, EduardCosmi, BenildePeiró, Ana M.Coughlan, GillianPelagalli, AlessandraCourtoy, Guillaume E.Pelidou, Sygkliti-HenriettaCretoiu, DragosPeng, GangCrimarco, AnthonyPeng, WenjingCristiani, Costanza MariaPenno, MeganCroft, BevinPérez-Herrero, María A.Cruz, Milagros Hidalgo De LaPergament, EugeneCytlak, UrszulaPerkovic, Matea NikolacDaimi, HouriaPetcherski, AntonDallio, MarcelloPetersen, MelissaDamborská, AlenaPeyré-Tartaruga, Leonardo AlexandreDamodaran, TirupapuliyurPezzolo, AnnalisaDas, Bibhuti BhusanPiccin, AndreaDash, Ranjeet PrasadPiciu, DoinaDatta, RupsaPickart, LorenDavies, Alun HPinto-Medel, MaríaDavies, Kay E.Pirtea, LaurentiuDavoudi, AnisPisanu, ClaudiaDawra, RajinderPisapia, PasqualeDe Jonge, HugoPiskin, SenolDe Luca, AntonellaPojskić, MirzaDe Paco Matallana, KatyPolańczyk, AndrzejDe Vito, DanilaPolano, MaurizioDegan, DianaPołap, DawidDella Gatta, FrancescoPosey, James ADemarquoy, JeanPreethichandra, Daluwathu Mulla GamageDevyatkin, VasiliyPrehn, Jochen H. M.Dhiman, KunalPricl, SabrinaDi Liddo, RosaPrimignani, MassimoDi Natale, ConcettaPrzybylowicz, KatarzynaDi Paolo, AntonelloPunetha, AnkitaDickinson, JoannePuthanveetil, PrasanthDioum, El Hadji M.Qi, Qian-RongDipaola, FrancaQian, ChunqiDivjak, EugenR. Shaker, MohammedDkhar, Hedwin KitdorlangRadisavljevic, ZivDobre, Robert-AlexandruRaffetto, JosephDocea, Anca OanaRahman, AteequrDockery, AdrianRaimundo, Armando Manuel De MendonçaDomagalska-Szopa, MalgorzataRakic, JelenaDong, FengpingRall, Katharina K.Donohue, Lee TRamadan, Qasem M.Dorfman, RuslanRamírez Sánchez, ManuelDoulamis, IliasRamkhelawon, BhamaDrain, PeterRamos, ErivanDuan, JialeiRamos-Petersen, LauraDubey, RaminRanstam, JonasDubrova, YuriRao, Rammohan V.Dugar, RohitRasool, SuhailDugar, Rohit P.Ratajczak, MonikaDuma, Virgil-FlorinRather, GulamDutta, PranabanandaRaya-González, JavierDziurkowska, EwelinaRaymond, JosetteEbensperger, GermánRaz, VeredEckstein, Max LennartRekaya, RomdhaneEdeas, MarvinRemijn, LianneEdelman, BradleyRengo, MarcoEfthymiou, StephanieRespino, MatteoEichhorn, Martin E.Reszka, EdytaEl Shamieh, SaidRevuri, VishnuElliott, EvanRibas-Maynou, JordiEmmersen, JeppeRicchiuto, PieroEnnequin, GaelRiccò, MatteoEnnis, EdelRivera, VictorEnomoto, HirayukiRizos, EmmanouilEpperla, NarendranathRoca, JosepErbel, RaimundRoceanu, AdinaEscolano-Pérez, ElenaRodrigues, Célia F.Escudero-Pérez, BeatrizRodriguez, AnnaEsfandyari, SaharRodríguez, Matías MorínEspadas Villanueva, IsabelRodriguez-Lafrasse, ClaireEspino, JavierRomeo, DomenicoEsposito, Maria TeresaRossi, AmerigoEstomba, Carlos Miguel ChiesaRossi, GemmaEsworthy, Robert StevenRossides, MariosEufemi, MargheritaRozerta, SokouFabrizio, Federico PioRubben, AlbertFaezipour, MisaghRückschloß, ThomasFalup-Pecurariu, OanaRuiz, Manuel J.Farrell, Philip M.Rüppel, JonasFassl, AnneSaba, LauraFatima, NidaSabloff, MitchellFeichtinger, MichaelSachmechi, IssacFeldo, MarcinSadée, WolfgangFeneberg, EmilySaenz De Viteri Vazquez, ManuelFeng, Hua-JunSaito, YuichiFeng, YunfeiSak, Jarosław J.Fernández De Las Peñas, CésarSakata, NaoakiFernandez, Bernadette O.Sakkas, Giorgos K.Fernandez, Hugo F.Saku, TakashiFernández-Revelles, Andrés B.Salamon, Katherine S.Ferrarazzo, GiuliaSalehzadeh-Yazdi, AliFerrari, ClarissaSamiotaki, MartinaFiedorowicz, EwaSandra, Lopez-LeonFigueras-Alvarez, OscarSandritter, Tracy L.Flannick, JasonSandy-Hodgetts, KylieFlores-Montero, JuanSantos, JerranForest, FabienSanz, Miguel A.Fotiou, DespinaSasagawa, MasaFountzilas, ChristosSatitsuksanoa, PattrapornFoutch, Brian K.Schaefer, BradleyFranchi, MatteoSchiavone, MarcoFrancuz, TomaszSchmutzler, AndreasFranklin, JanetSchuchardt, MirjamFrenkel, AmitSchultz, StephenFriedrichs, NicolausSchumm, Walter R.Froelich, Matthias F.Scicchitano, PietroFroyen, GuyScioscia, CrescenzioFujimori, NaoScomparin, AnnaFujita, MitsuguScoppola, AlessandroFujiwara, KenjiSeguí-Ripoll, José MiguelFukai, JunyaSellers, DianeFukumoto, YoshihiroSenbonmatsu, TakaakiFulda, Kimberly GSepuru, Krishna MohanFuruta, SaoriSerafini, GianlucaGajdzis, PawelSerralheiro, PedroGalli, AlavaroSetty, BhuvanaGallo, AlessiaSfondrini, Maria FrancescaGallo, GaetanoShah, JainilGallyas, FerencShah, RaviGaman, Mihnea-AlexandruShan, Zack YGambardella, JessicaShankar, EswarGarcia Perez, SamuelSharma, UmakantGarcía-Redondo, AlbertoSheng, KuanweiGayoso-Cabada, JoaquínSherratt, R. SimonGaze, Mark N.Shibata, YasushiGelissen, IngridShibukawa, GoroGentile, EleonoraShidal, ChrisGerke, OkeShin, DongheeGhag, GauravShmatov, MikhailGhanbarzadeh-Dagheyan, AshkanShort, MichalaGhidini, MicheleShui, Hao-aiGhita, MihaelaShukla, Surendra KumarGiacobini, MaiBrittSiegel, Tiffany PortaGiannattasio, CristinaSilva, CatarinaGiannotti, ElisabettaSilva, MihiriGiliberto, LucaSirbu, OvidiuGill, Pritmohinder S.Skladany, LubomirGiovanetti, AnnaSkonieczna-Żydecka, KarolinaGiri, HemantSkuja, SandraGlotov, Andrey S.So, JonathanGogulamudi, Venkateswara ReddySokol, ElizabethGoh, Gerard Kian-MengSokolis, Dimitrios P.Golubnitschaja, OlgaSong, DongGood, Deborah J.Song, HailongGoyanes, AlvaroSoukupova, JanaGrabarek, Beniamin OskarSowa, IreneuszGraf, Solomon A.Sperber, NinaGrammatikopoulou, Maria G.Srivastava, AkritiGraslund, TorbjornStachowska, EwaGrelet, SimonStanciu, Gabriela-DumitritaGrossi, ValentinaStark, Amy L.Gu, FengStark, ChristinaGuerini, Franca R.Stavinoha, Peter L.Gunnarsson, UlfSteinbrunn, TorstenGupta, RisheinStewart, MarkGutiérrez-Colosía, Mencía R.Štrac, Dubravka ŠvobHa, JeonghoonStraccia, PatriziaHadano, ShinjiSubudhi, SonuHain, DebraSuciu, Bogdan AndreiHakovirta, Harri H.Sun, LuHan, ChunhuiSung, Wen-WeiHan, MingzhiSuryavanshi, Santosh V.Harris, David T.Suwan, KeittisakHatano, YuichiroŚwiątkiewicz, IwonaHauser, Alexander SebastianSzczepanek, JoannaHayashi, RyujiSzczerbinski, LukaszHe, FengTagliapietra, MatteoHeakal, YasserTakatani-Nakase, TomokaHeber, StefanTakebayashi, ShigeoHeijs, BramTaked, TakayukiHendricks-Sturrup, Rachele M.Tamaian, RaduHenrich, DirkTamura, YasuhisaHettich, GeorgTan, YutingHeyman, BenjaminTanaka, Hiroyoshi Y.Higaki, AkinoriTandon, RiteshHipsley, AnnMarieTanini, DamianoHizel, CandanTashima, ToshihikoHokamp, Nils GroBeTeixidó, CristinaHomma, TetsuyaTelyshev, DmitryHong, In-SunTempl, MatthiasHorne, CharlotteTerreehorst, IngridHortobagyi, TiborThalamuthu, AnbupalamHosseinpour, SepantaThandra, Krishna ChaitanyaHouse, CarrieThanigaimani, ShivshankarHuang, JijunThavamani, AravindHuang, XudongThierry, AlainHuck, OlivierThomas, ThresiaHudlikar, RasikaThomssen, ChristophHung, KuofengTimpani, CaraIacoangeli, AlfredoTing, Wen-ChienIbrahim, FandiTitz, BjoernIchikawa, HajimeTobias Sejbaek, MathiesenIerardi, EnzoToedebusch, ChristineIlewicz, GrzegorzToelle, BrettInfante, ArantzaToillon, Robert-AlainInzani, FredianoToma-Fukai, SachikoIorga, MagdalenaTomasik, BartłomiejIshige, TakashiTomaszkiewicz, MartaIshikawa, YuichiTong, MingmingIslam, Md SorifulToniolo, LuanaIssa, SamarTorras-Ambròs, JoanIwasaki, JuaTorres-Sánchez, IreneIyer, Arun KToshimitsu, YamaokaIyer, JanakiTota-Maharaj, RajeshIzawa, Kazuhiro P.Tourkmani, Abdo KarimJacobs-Cachá, ConxitaTrapani, DarioJäger, WalterTrentini, AlessandroJähn, KatharinaTrepanier, AngelaJahrami, HaithamTriaca, VivianaJamal, LeilaTritapepe, LuigiJaneh, OmarTrivanovic, DrenkaJanic, AnaTroy, Niamh MJankovic, MomciloTsai, Han-MouJansone, BaibaTsampras, NikolaosJarcuska, PeterTSUJI, DaikiJavid, MahsaTuffery-Giraud, SylvieJavier Jiménez-Jiménez, FelixTumedei, MargheritaJenkins, Samir V.Tupler, RossellaJennifer, RabinTural, ÜmitJi, HongUllah, AminJin, XinUllah, ZahidJohnson, DanielleUrbaniak, AlicjaJohnson, Jay E.Urbano, Paulo César MartinsJorgensen, AndreaUssowicz, MarekJovel, JuanVaismoradi, MojtabaKaina, BerndValles, Soraya L.Kajtár, BélaVan Der Bent, M. LeontienKalaska, BartlomiejVan Der Heijden, JeroenKällback, PatrikVan Dort, Christa J.Kanda, TatsuoVarrassi, GiustinoKandasamy, PalanivelVassiliou, Aliki GeorgiaKartsonaki, ChristianaVecellio, MatteoKashiwagi, ShinichiroVemuri, RavichandraKaskel, Frederick J.Venneri, Mary AnnaKasprzak, Magdalena PaulinaVerma, NirmalKassassir, HassanVetra, AnitaKassi, MahwashVidal, ReneKasuga, KensakuVignes, MichelKataoka, HiroshiVila-Suárez, Maria ElenaKatrinli, SeymaVitale, Salvatore GiovanniKatuchova, JanaVladimirov, VladimirKawabata, HideakiVoellger, BenjaminKayser, KlausVousoura, EleniKędzierska, EwaVoyer, JohnKhaddaj Mallat, RayanVulturar, RomanaKhalaf, FatimahVuorio, AlpoKhan, Mohd MohsinWalecki, PiotrKhan, Muhammad SaadWang, HuaishanKim, Hye RyounWang, KaiKim, Ju HanWang, MaosenKim, KaWang, Thomas D.Kim, Raymond HWang, YumengKim, So-YounWarzecha, ZygmuntKim, Tae HyunWas, Christopher AKinoshita, ChisatoWatanabe, YoichiKitai, TakeshiWen, Yu-ChingKnepper, ToddWestergaard, NielsKo, Hsin-ChiWhite, Stormi PulverKobeissy, FirasWhitelegge, JulianKobierzycki, ChristopherWiest, Michael C.Kodidela, SunithaWilczyński, Jacek R.Komnenov, DraganaWilhelm, ThereseKoper-Lenkiewicz, Olga MartynaWillemse, ElineKosinsky, Robyn LauraWilliams, JanetKotsilieris, TheodoreWilliams, Stephen J.Kouji, HaradaWilm, MatthiasKouokam, Joseph CalvinWilton, SteveKoyuncu, SedaWirth, MarkusKozieł, MonikaWisniewski, AdamKrajewska-Włodarczyk, MagdalenaWolińska, EwaKroeze, Stephanie G.C.Wolter, TilmanKucukseymen, SelcukWong, YUjunKukull, Walter A.Wood, Andrew JamesKulkarni, Krishnaji R.Wu, Alan H.Kumar, VijayWu, Lawrence Shih HsinKuo, Chao-HungWu, Tzong-YuanKuwata, ShingoWuebbles, RyanKwok, Hoi-HinXue, XiangLa Starza, RobertaYamaoka, ToshimitsuLachowicz, Joanna IzabelaYamashita, KentaroLagundzin, DraganaYamashita, ShinjiLahiri, SatadruYan, ZiyingLambrou, George IYasu, TakanoriLandon, Chelsea DawnYates, Nathanael J.Lanza, GiuseppeYeh, I-JuLanzone, JacopoYen, Ju-YuLao, OscarYip, Bon HamLarson-Prior, Linda J.Yoshii, IchiroLatini, AndreaYu, YanboLawson, AdamYunusa, IsmaeelLecerf, Jean-michelZacharias, Helena U.Ledesma, Maria DoloresZago, VanessaLee, Jae-GilZammit, AndreaLee, Li-JenZandt, Rene In ‘tLee, Sang BongZaucha, JanLee, Yee MingŽdralević, MašaLehto, MikaZegan-Barańska, MałgorzataLewis, ChandaniZeineldin, MohamedLi, ChenyuZhang, KeqiangLi, Chien-FengZhang, WeijieLi, HuaZhang, WencaiLi, Ruei-NianZhao, BaoyinLi, Shengwen CalvinZhao, YuLiang, LiangZhou, QingyuLiao, XinqinZou, ChunbinLin, Chung-YingZurth, Christian

